# Association between oesophageal cancer and biomass smoke exposure: a case-control study

**DOI:** 10.3332/ecancer.2022.1422

**Published:** 2022-07-04

**Authors:** Violet Kayamba, Chola Mulenga, Malambo Mubbunu, Lydia Kazhila, Phoebe Hodges, Paul Kelly

**Affiliations:** 1 Tropical Gastroenterology and Nutrition Group, University of Zambia School of Medicine, PO Box 50398, Lusaka, Zambia; 2 Blizard Institute, Barts & The London School of Medicine and Dentistry, Queen Mary University of London, 4 Newark Street, London E1 2AT, UK; a https://orcid.org/0000-0002-6521-2501

**Keywords:** oesophageal cancer, Zambia, biomass, risk factors

## Abstract

Most African populations are regularly exposed to biomass smoke, but knowledge of associated health implications is limited. This study aimed to investigate the association between oesophageal cancer (OC) and exposure to biomass smoke. This case-control study was conducted in Lusaka, Zambia. Cases were patients with endoscopically diagnosed OC, while controls were healthy volunteers. Questionnaires were used to collect lifestyle risk factors. Two sets of data were analysed; one with unmatched cases and controls and the other one with matching by age and sex. We enrolled 366 patients (131 cases and 235 controls). Among the cases, 50 (38%) were female and the median age was 56 years (IQR = 46–65 years). OC was significantly associated with domestic exposure to biomass smoke in univariate analysis (OR: 3.1; 95% CI: 1.7–5.6, *p* < 0.001) and after adjusting for potential confounders (OR: 2.1; 95% CI: 1.1–3.8, *p* = 0.017). Matched comparisons showed similar results for this association in univariate analysis (OR: 2.9; 95% CI: 1.5–5.8, *p* < 0.001) and using conditional logistic regression (OR: 2.8; 95% CI: 1.3–5.9, *p* = 0.005). Other risk factors found to be associated with OC were rural residence (OR: 2.3; 95% CI: 1.0–5.3, *p* = 0.004), lack of formal education (OR: 3.9; 95% CI: 1.5–9.9, *p* = 0.04) and living in poor housing (OR: 2.4; 95% CI: 1.1–5.6, *p* = 0.034). In conclusion, there is an association between OC and domestic exposure to biomass smoke and other lifestyle factors linked to low socio-economic status.

## Introduction

In 2020, 604,100 oesophageal cancer (OC) cases and 544,076 deaths were recorded worldwide [1]. The two main histological subtypes of OC are oesophageal squamous cell carcinoma (OSCC) and adenocarcinoma [2]. About 80% of the OSCC cases occur in low- and middle-income countries. Definite risk factors of OSCC are tobacco and alcohol with evidence for the others, including poor oral hygiene, caustic injury to the oesophagus and radiation [3], which is still being investigated. Recently, there has been growing evidence implicating the consumption of hot beverages as a risk factor for OSCC [4–6]. On the contrary, obesity and Barrett’s oesophagus are significant risk factors for oesophageal adenocarcinoma [7].

There are clear geographical variations in OSCC incidence across Africa [8, 9]. The highest incidence of OSCC in Africa is along the eastern corridor—extending from Ethiopia to parts of South Africa [10]. The OSCC epidemiological variation across Africa justifies the need for further aetiological research within individual countries. There is growing evidence linking lifestyle and environmental factors [10–12] in OSCC pathology across Africa. In addition, recent studies have explored the role of genetics on OSCC development in Africa, such as that by Moody *et al* [13].

While biomass smoke has not yet been confirmed on the list of definite OC [14] risk factors, we recently reported a significant association between increased OSCC risk and biomass smoke exposure [15]. Exposure to biomass smoke is very common in Africa with over 70% of its population relying on biomass fuels [16]. Biomass smoke constitutes a complex mixture of compounds, some of which are similar to known tobacco smoke-related carcinogens [17]. It is therefore possible that biomass smoke could be influencing carcinogenesis using similar mechanisms to cigarette smoke.

This study aimed to investigate the influence of biomass smoke exposure (in connection with other risk factors) on OC development using a case-control study approach.

## Methods

This hospital-based case-control study was conducted at the University Teaching Hospital (UTH) in Lusaka, Zambia, between October 2018 and May 2021. UTH is the largest referral hospital in Zambia. OC patients above the age of 18 years, presenting to the UTH endoscopy unit for upper gastrointestinal endoscopy were considered for enrolment. Healthy controls were enrolled among caregivers and other asymptomatic volunteers within the UTH. They were above 18 years and without any obvious health condition. All study participants gave written consent. We excluded individuals with prior history of cancer diagnosis or therapy. This study was performed in line with the principles of the Declaration of Helsinki. Approval was granted by the University of Zambia Biomedical Research Ethics Committee (ref. no. 001-11-17).

### Study procedure

#### Enrolment of study participants

All patients with oesophageal lesions suspected to be malignant on endoscopy (during the study period) were requested to participate as cases. During the endoscopy, at least six biopsies were collected from the lesions in accordance with standard of care. These were fixed in 10% neutral buffered formalin for histopathological processing. Slides were stained using routine haematoxylin and eosin to facilitate histological diagnosis of OC subtypes. Enrolled as controls were healthy volunteers who did not have any gastrointestinal symptoms. In addition, we also enrolled controls from among persons who had escorted relatives for endoscopy. We used interviewer-administered questionnaires to collect information on OC risk factors. Included in the questionnaire were demographic, medical history, lifestyle and OC environmental risk questions.

#### Exposure assessment

To determine exposure to biomass smoke, we asked study participants about the source of fuel used for cooking, heating and lighting in their homes. Those who admitted to being reliant on firewood, charcoal, dung or grass were categorised as having exposure to biomass smoke. We collected details on housing from the participants, including the type of roofing, wall, number of rooms and cooking areas. We also asked about the availability of basic items such as television sets, fridges, computers, microwave ovens, cars, etc. We gathered information on whether or not the water sources to participants’ households were piped. Study participants were asked about a history of eating soil (geophagia). The same questionnaires also sought a history of alcohol consumption and tobacco use.

### Data analysis and sample size calculation

To calculate the required sample size, we used previously published work [14]. We found that 68% of the OC patients and 38% of the endoscopic controls were regularly exposed to biomass smoke in their homes. At 90% power and an alpha level of 0.05, at a 1:1 proportion of cases to controls, we needed at least 63 cases and 63 controls.

Data were analysed using Stata version 15. Continuous variables were summarised using median and interquartile ranges as the data were non-parametric when tested using the Shapiro–Wilk test. Percentages were used to summarise categorical variables. Analysis was carried out for both matched and unmatched cases and controls. Matching was done by sex and age. McNemar’s test was used for matched data and Fisher’s exact test for unpaired data. In order to adjust for potential confounding, conditional logistic regression for matched and unconditional logistic regression for unmatched data was used. A two-sided alpha value of 0.05 at a confidence level of 95% was considered a threshold for statistical significance.

## Results

### Basic demographic characteristics of oesophageal cancer patients enrolled in the study

We enrolled a total of 366 participants, of which 131 were cases and 235 were controls. Among the cases, 50 (38%) were female and the median age was 56 years (IQR = 46–65 years). The majority of OC patients (88%) were from Lusaka, eastern, central and southern provinces (Table 1). Histopathology reports were available for 100 OC patients, and of these, 89 (89%) were squamous cell carcinoma and 11 (11%) were adenocarcinomas. For the remaining 31, histological confirmation was made at private facilities with a similar proportion of histological sub-types.

### Risk factors for oesophageal cancer

We analysed the occurrence of risk factors by comparing cases and controls. In univariate analysis, exposure to biomass smoke, poor housing, lack of basic household goods, lack of formal education and cigarette smoking were associated with increased odds of OC (Table 2). Analysis of matched cases and controls revealed that exposure to biomass smoke, rural residence, cigarette smoking, poor housing, lack of basic household goods and formal education were risk factors for OC in Zambian patients, as shown in Table 2. Use of charcoal (OR: 2.4; 95% CI: 1.4–4.5, *p* = 0.001) or firewood (OR: 3.9; 95% CI: 1.8–8.3, *p* < 0.001) was each associated with OC.

We ran three different models using stepwise logistic regression of unmatched cases and controls. Model 1 included all the variables analysed in univariate analysis. In model 2, we removed variables less than 300, as that reduced the total numbers and could have introduced some biases. Model 3 was run on variables related to the environment. The first model showed that age more than 45 years (*p* = 0.009), rural residence (*p* = 0.002), lack of formal education (*p* = 0.035) and tobacco smoking were associated with OC, while in the second and third models, biomass smoke exposure showed a statistically significant association (Table 3).

For matched cases and controls, we used conditional logistic regression. The same variables were used for models 1, 2 and 3 as described above. Exposure to biomass smoke was significantly associated with OC in models 2 and 3 with OR: 2.8, 95% CI: 1.3–5.9, *p* = 0.005 and OR: 2.4, 95% CI: 1.2–4.5, *p* = 0.016, respectively.

### Domestic fuel use and oesophageal cancer

The main fuels used by study participants included electricity, charcoal, firewood, kerosene, gas and solar. Overall, 89 (92%) rural residents relied on biomass fuels compared to 165 (63%) urban dwellers. A comparison of the cases and controls showed significant differences in the fuel used for cooking (*p* < 0.001), lighting (*p* = 0.02) and heating (*p* = 0.003) (Table 4). These effects remained significant after adjusting for cooking and sleeping areas within the household.

## Discussion

OC is a growing problem in south-eastern Africa, with several aetiological factors implicated. This study has established biomass smoke exposure as a possible risk factor, which is consistent with previous findings from Zambia and the surrounding regions [15, 18, 19]. We further report that residing in a rural area, lack of formal education and poor housing are also risk factors for OC.

The World Health Organisation reports that household air pollution, including biomass smoke exposure, increases the risk of developing numerous lethal and chronic conditions, such as heart diseases [20]. Biomass smoke exposure is very common in many parts of the world, with over 70% of the African population regularly exposed [16]. Previously, we presented preliminary data linking biomass fuel use with increased risk of oesophageal and stomach carcinogenesis [15, 21]. An Iranian prospective cohort study of up to 50,045 participants reported that household burning of biomass fuels increased the risk of digestive tract cancers among individuals [22]. There is an urgent need for experimental studies (involving both human and animal models) to identify internal dose carcinogenic biomarkers. In an area of high OC prevalence in south-west Kenya, significantly higher urinary polycyclic aromatic hydrocarbons (probable carcinogens) were established [23].

Cancer development is a complex process, with several factors acting synergistically. We explored multiple lifestyle factors and found a significant association between proxies of poverty and OC risk. This finding is common elsewhere, where it is shown that individuals from the low socio-economic class than the higher socio-economic class are more prone to developing OC risk than the latter [24]. It is not clear which poverty-driven factors lead to OC, but pollution compounded by poor ventilation might be a contributing factor.

Similar to our previous findings, we failed to demonstrate a link between alcohol intake and OC. This could result from the data collection approach in which we relied on recall and categorised individuals into two: those who drank alcohol and those who did not. This approach did not allow us to establish a threshold amount of alcohol intake responsible for OC increased risk. We, however, found that cigarette smoking was an independent risk factor in unmatched comparisons. Cigarette smoking is a well-established risk factor for OC [25], but with varying molecular evidence. In a similar population to ours, whole-exome sequencing and RNA transcriptomic analysis of 59 Malawian OC patients did not find any signatures of tobacco smoking [26], while mutational signatures for tobacco were reported in a study that included samples from 8 countries, including East Africa [13].

Africa has had a relatively low cancer research output, with most of its publications coming out of South Africa and Egypt [27]. With limited outputs from countries most heavily affected by OC, risk factors that could be affecting African populations have not been thoroughly investigated. The need for intensifying cancer research in Africa is an urgent one. In addition to understanding risk factors, there is a need to enhance data collection on treatment outcomes and streamline where the intention is curative or palliative [28]. Early OC detection offers the best chance at good outcomes, but unfortunately this seldom occurs, and in Africa, such information is not well documented or reported.

From our sample size calculations, we enrolled enough study participants to allow us draw evidence-based conclusions. However, a major limitation was the lack of an objective way to measure biomass smoke exposure. Still, we based our assessment purely on household use of biomass fuel. In future, we intend to institute objective measurements using wearable devices to determine exposure more accurately.

## Conclusion

There is an association between OC and biomass smoke exposure. Therefore, minimising exposure could potentially impact the growing burden of OC in Zambia. Moreover, there is a need to investigate how other factors (lack of formal education, suitable housing and rural residence) interact with biomass smoke in OC pathogenesis.

## Declarations

Not applicable.

## Funding

None.

## Conflicts of interest

The authors have no conflicts to declare.

## Data availability

Data will be available from the corresponding author upon reasonable request

## Authors’ contributions

VK and PK designed and conceptualised the study. VK, CM, MM and PH enrolled the study participants and collected the data, VK, LK and PK analysed the data. All the authors were involved in manuscript writing and approval of the final version.

## Figures and Tables

**Table 1.** Basic characteristics of the oesophageal cancer patients included in the study.

Characteristic	Number of patients Overall *n* = 131	Proportion
**Age:** Less than 45 years 45–60 years Above 60 years Missing	29 46 47 9	22% 35% 36% 7%
**Sex:** Female Male	50 81	38% 62%
**Residence:** Urban Rural Missing	84 45 2	64% 34% 2%
**Province of residence:** Lusaka Central Eastern Southern Copperbelt Northern Western Muchinga North-western Luapula Missing	65 18 16 16 6 5 2 1 1 0 1	50% 14% 12% 12% 5% 4% 2% 0.8% 0.8% 0% 0.8%
**Education level attained:** Tertiary Secondary Primary None	10 17 51 51	7% 13% 40% 40%
**Occupation:** Employed by government Employed in the private sector Farmer Self-employed (other than farming) None Missing	15 33 22 36 16 9	12% 25% 17% 27% 12% 7%
Body mass index (kg/m^2^): Underweight (<18.5) Normal (18.5–24.9) Overweight (25.0–29.9) Obese (>29.9) Missing	58 22 5 0 46	44% 17% 4% 0% 35%

**Table 2.** Univariable analysis of risk factors for oesophageal cancer.

Risk factor	Cases *n* = 131 (%)	Controls *n* = 235 (%)	Univariate OR (95% CI)	*p*-value	Cases *n* = 118 (%)	Controls *n* = 118 (%)	Univariate OR (95% CI)	*p*-value
	**Unmatched**				**Matched by age and sex**			
Female	50 (38)	131 (56)	0.5 (0.3–0.8)	**0.002**				
Less than 45 years	26 (24)	93 (41)	0.5 (0.3–0.9)	**0.003**				
Low BMI^a^ (kg/m^2^)	57 (67)	11 (5)	35 (16–82)	**<0.001**	51 (56)	7 (7)	9.3 (3.3–35.7)	**<0.001**
Biomass smoke exposure	108 (84)	150 (64)	3.1 (1.7–5.6)	**<0.001**	97 (84)	73 (62)	2.9 (1.5–5.8)	**<0.001**
Rural residence	45 (35)	52 (22)	1.9 (1.1–3.1)	**0.013**	42 (36)	27 (23)	1.8 (1.0–3.3)	0.053
Poor housing	40 (35)	42 (18)	2.4 (1.4–4.2)	**0.001**	36 (36)	20 (17)	3.3 (1.4–8.3)	**0.003**
Lack of basic household goods	68 (55)	83 (35)	2.3 (1.4–3.4)	**<0.001**	61 (56)	44 (37)	2.3 (1.2–4.3)	**0.008**
Lack of formal education	51 (40)	52 (22)	2.3 (1.4–3.8)	**0.001**	47 (41)	24 (20)	4.0 (1.8–10.1)	**<0.001**
Cigarette smoking	39 (36)	29 (14)	3.4 (1.9–6.2)	**<0.001**	36 (37)	23 (23)	1.9 (0.9–4.4)	0.11
Alcohol consumption	47 (41)	71 (33)	1.4 (0.9–2.3)	0.15	43 (41)	45 (42)	1.0 (0.5–2.0)	1.00
Consumption of hot drinks	99 (76)	173 (74)	1.1 (0.7–1.9)	0.71	86 (73)	86 (73)	1.00 (0.5–1.8)	1.00
Piped water source	88 (67)	171 (73)	0.7 (0.5–1.2)	0.23	80 (68)	78 (67)	1.0 (0.6–2.0)	1.00
Geophagia	9 (7)	31 (13)	0.5 (0.2–1.1)	0.08	7 (6)	13 (11)	0.6 (0.1–1.6)	0.33

^a^ BMI is body mass index which considered low if less than 18.5 kg/m^2^

*p*-values in bold were statistically significant

**Table 3.** Logistic regression models evaluating risk factors for oesophageal cancer.

Unmatched cases and controls (unconditional)			Matched cases and controls (conditional)		
Model 1*	**OR (95% CI)**	***p*-value**	Model 1*	**OR (95% CI)**	***p*-value**
Age less than 45 years	0.4 (0.2–0.8)	0.009	Lack of formal education	2.9 (1.1–7.5)	0.032
Lack of formal education	2.0 (1.0–3.7)	0.035	Rural residence	2.3 (1.0–5.3)	0.040
Rural residence	2.7 (1.5–5.0)	0.002			
Smoking tobacco	2.9 (1.5–5.7)	<0.001			
Model 2**	**OR (95% CI)**	***p*-value**	Model 2**	**OR (95% CI)**	***p*-value**
Age less than 45 years	0.5 (0.3–0.9)	0.017	Biomass smoke exposure	2.8 (1.3–5.9)	0.005
Sex – female	0.4 (0.2–0.7)	0.001	Lack of formal education	3.9 (1.5–9.9)	0.004
Biomass smoke exposure	2.1 (1.1–3.8)	0.017			
Lack of formal education	2.5 (1.4–4.4)	0.001			
Model 3***	**OR (95% CI)**	***p*-value**	Model 3***	**OR (95% CI)**	***p*-value**
Age less than 45 years	0.4 (0.3–0.8)	0.004	Biomass smoke exposure	2.4 (1.2–4.5)	0.016
Sex – female	0.5 (0.3–0.8)	0.004	Lack of good housing	2.4 (1.1–5.6)	0.034
Biomass smoke exposure	2.0 (1.1–3.7)	0.019			
Lack of good housing	2.1 (1.2–3.8)	0.009			
*Model 1: Sex, age, biomass smoke exposure, poor housing, lack of household goods, rural residence, lack of formal education, current smoker, alcohol intake, consumption of hot beverages, lack of piped water and geophagia.			*Model 1: Biomass smoke exposure, poor housing, lack of household goods, rural residence, lack of formal education, current smoker, alcohol intake, consumption of hot beverages, lack of piped water and geophagia.		
**Model 2: Sex, age, biomass smoke exposure, poor housing, lack of household goods, rural residence, consumption of hot beverages, lack of piped water and geophagia.			**Model 2: Biomass smoke exposure, poor housing, lack of household goods, rural residence, consumption of hot beverages, lack of piped water and geophagia.		
***Model 3: Sex, age, biomass smoke exposure, poor housing, lack of household goods and rural residence.			***Model 3: Biomass smoke exposure, poor housing, lack of household goods and rural residence.		

**Table 4.** Association between oesophageal cancer and type of energy used for cooking, lighting and heating in homes.

	Cooking		Lighting		Heating	
	Cases, *n* = 131 *n* (%)	Controls, *n* = 234 *n* (%)	Cases, *n* = 127 *n* (%)	Controls, *n* = 226 *n* (%)	Cases, *n* = 77 *n* (%)	Controls, *n* = 154 *n* (%)
Electricity	22 (17)	82 (35)	65 (51)	149 (66)	5 (6)	12 (8)
Charcoal	75 (57)	116 (50)	12 (9)	11 (5)	35 (45)	100 (65)
Firewood	33 (25)	34 (15)	9 (7)	7 (3)	28 (36)	37 (24)
Kerosene	1 (1)	34 (15)	2 (2)	0 (0)	7 (9)	2 (1)
Gas	0 (0)	0 (0)	0 (0)	0 (0)	1 (1)	0 (0)
Solar	0 (0)	1 (0.5)	24 (19)	40 (18)	1 (1)	3 (2)
Candles	-	-	15 (12)	19 (8)	0 (0)	0 (0)
*p-value	<0.001		0.02		0.003	
